# What’s in a word? The framing of health at the regional level: ASEAN, EU, SADC and UNASUR

**DOI:** 10.1177/1468018115599816

**Published:** 2015-12

**Authors:** Ana B Amaya, Vincent Rollet, Stephen Kingah

**Affiliations:** United Nations University Institute on Comparative Regional Integration Studies, Belgium; Wenzao Ursuline University/French Center for Research on Contemporary China (CEFC), Taiwan; United Nations University Institute on Comparative Regional Integration Studies, Belgium

**Keywords:** Foreign policy, health policy, policy frames, regional organisations

## Abstract

The Association of Southeast Asian Nations, the European Union, the Southern African Development Community and the Union of South American Nations have increasingly been involved in health diplomacy in the past decade, yet little is known about how they frame health as a foreign policy issue and how this has an impact on their prioritisation of policies. For this, we conducted a review of existing grey and peer-reviewed literature that address regional integration and health, as well as a documentary review according to security, development, trade, human rights, moral/ethical reasonings and global public goods frames identified in the literature. The policy frames identified responded to the challenges these regions currently face. The Association of Southeast Asian Nation’s struggle with re-emerging diseases has led to favouring a securitisation approach to health, the European Union approaches health as a cross-cutting policy issue, the Southern African Development Community presents health as a driver for development, and while the Union of South American Nations emphasises health as a human right and addresses the social determinants of health as an ethical imperative. Overall, these policy frames were useful in analysing the framing of health in foreign policy at the regional level. However, within our analysis, we identified a new frame that approaches health as an intersectoral issue. The impact of regional organisations’ forward will depend on their ability to harness their convening power and speak in a coherent voice on health matters.

## Introduction

There is an increasing call for multilevel governance in health (Ottersen et al., 2014) and greater understanding of how health can be protected and promoted within various global processes (Ottersen et al., 2011). Within multilevel governance for health, supra-national bodies that oversee country health commitments, generate common positions and facilitate joint collaboration to reach health targets, emerge as critical (Deacon, 2005; Kaasch and Stubbs, 2014). Increasingly, regional organisations are carving a place in negotiating health targets. Health has become a concern for these organisations given the challenges that arise from diseases that transcend borders; the ability to respond to issues such as the migration of health workers and collaborating on health infrastructure; as well as using health activities as a way to strengthen links between member states (Amaya et al., 2015).

Indeed, there is increasing appreciation that health is an effective tool to achieve foreign policy goals compared to other forms of international development using health programmes to improve the influence and security of donor countries and organisations or as a manner to pursue non-health objectives (Kevany, 2014). This has led to what has been termed ‘global health diplomacy’, where the alignment of actor interests towards health has generated opportunities for international partnerships (Fidler, 2007; Kevany, 2014). We argue that regional organisations can also serve as a space for countries to position themselves in the multilateral arena through what can be termed ‘regional health diplomacy’. This has been explored to a degree for South America, echoed in the work of Riggirozzi (2015) but as this article shows, this is prevalent in other regions as well. This term and the definition of regional health policies will be discussed in greater detail in an upcoming article.

As blocs that interact with other organisations at the global level, regional organisations’ positions on health diplomacy are frequently non-binding. However, these common positions provide an opportunity to harness individual country strengths towards common health goals (Riggirozzi, 2014), as well as increase the influence of the organisation globally. Consequently, it is important to understand how regional bodies develop arguments on health diplomacy. Narratives of health within policy making and foreign policy engagement are becoming a distinctive issue in health governance. Comprehending actors’ positions on health can potentially lead to a more transparent policy dialogue in decision-making that affect health as well as the possibility to hold actors accountable to commitments made (Ottersen et al., 2014).

Moreover, the literature shows that framing these narratives can be instrumental in introducing an issue into political agendas potentially leading to the institutionalisation of norms (Boas and McNeill, 2004; Finnemore and Sikkink, 1998). For instance, the Millennium Development Goals (MDGs) are an example of an initiative that emerged from a norm regarding poverty as morally unacceptable and something that should generate collective action for its eradication (Fukuda-Parr and Hulme, 2009; Ottersen et al., 2014).

Several authors have sought to research the relationship between health and foreign policy. One study found that health in foreign policy can be addressed as a security issue, charity, investment or public health more generally (Stuckler and McKee, 2008). Another study identified four main discourses on health governance as biomedicine, economism, human rights and security (Hill, 2011). On the other hand, Kickbusch (2011) categorises the links between health and foreign policy using economic, security and social justice terms. According to this latter view, health is prioritised in foreign policy driven by the fear of global pandemics or the intentional spread of pathogens (security); the economic effect of poor health on development (economic); or by reinforcing health as a social value and human right (social justice).

However, within this literature researching the framing of health policy, there is a significant lack of understanding of how regional organisations address health, in part due to the recent incursion of some of these organisations in this area. While the international relations literature has addressed regionalism for some time now, research on the social approach of these regional organisations is relatively incipient. With this research, we build on the New Regionalism Approach (NRA)^1^ by developing new frameworks that explain ‘regionness’ and social cohesion beyond the traditional areas of study of regions of trade and security, to socio-political areas such as health (Hettne and Soderbaum, 1998). We contribute to the existing debate, providing greater insight into narratives of health as an area of regional governance that has been relatively unexplored in the literature.

In this study, we employ critical discourse analysis by using the policy frames identified by Labonte and Gagnon (2010). The authors identify security, development, global public goods (GPG), trade, human rights and ethical/moral reasoning as the most common ways in which countries approach global health in their foreign policy. They do this by examining several key government policy documents and find that although these governments are committed to health as a foreign policy goal, decisions are primarily made on the basis of national security and economic interests. According to them, while development, human rights and ethical/moral reasoning are present in the discourse, they rarely dominate practice.

We take this a step further and seek to understand if these same policy frames apply at the regional level or if new policy frames emerge. What does this explain about how these regions address health problems and what are the implications of this? We do this by examining key documents that address health in four regional organisations representing four different continents: the Association of Southeast Asian Nations (ASEAN), the European Union (EU), the Southern African Development Community (SADC) and the Union of South American Nations (UNASUR).

The article follows with a methods section on literature review and documentary analysis. This precedes a results section that presents the main findings from our four regions under study, a discussion of these findings and final conclusion.

## Recasting narratives of health

This article seeks to understand the narratives of framing health as a driver of foreign policy at the regional level. For this purpose, we analyse the documents according to the policy frames developed by Labonte and Gagnon (2010), as well as definitions developed by other authors in the field (see Table 1). We chose this framework for our analysis for several reasons. Unlike other existing frameworks, their policy frames were the result of a thorough examination of foreign policy documents at the governmental level and not the global health governance level. This means the framework is not only based on the examination of existing foreign policies, but the results emerge from country views, some of which belong to our regions of study. It was also revealing to note that their research found that decisions in foreign policy at the governmental level were primarily based on the ‘high politics’ of national security and economic concerns rather than the traditional ‘low politics’ of foreign policy as pertains to development, human rights and ethical/moral arguments. This raises the following question: Is this also the case at the regional level? In addition, the six policy frames are comprehensive enough allowing evaluation of the different perspectives of health included within the policy documents.

**Table 1.** Conceptualisation of health and foreign policy frames.

Policy frame	Conceptualisation	Arguments from Labonte and Gagnon (2010)	Examples of key words
1. Security	‘Health framed as a traditional security issue emphasises the defence of borders against infectious diseases and bioweapons with little consideration for non-communicable diseases and social determinants of health’ (Ng and Ruger, 2011)	*‘* ***Security*** gives global health interventions greater traction across a range of political classes than a rights-based argument alone. To the extent that this strengthens a base of public health expansion, securitisation of health may be a prerequisite to its eventual de-securitisation. But vigilance is needed to avoid national security from trumping human security’.	Security; securitisation; conflict; defence; stability; political; pandemics; protection.
2. Development	‘Health is key to development and combating poverty. Hunger is a major cause of ill health. Structural causes of poverty and hunger are interwoven, and part of a nexus of policies where foreign policies also play an important part’. (Amorim et al., 2007)	*‘* ***Development*** remains the invitation to global governance debates. It provides a seat at the table. Risks inherent in its ‘investing in health’ instrumentalism can be tempered by continuously reminding decision makers to distinguish which one is the objective (human development) and which one the tool (economic growth)’.	Development; human development; poverty; economy; neglected population; equity; aid; support.
3. Global public goods	‘The framing of health as “commons” or as a “global public good” conceives of health as something beyond the jurisdiction of any one country and of interest to two or more countries or their populations. Public goods are non-excludable and non-rival –people cannot be excluded from consuming such goods, nor does one person’s consumption of such goods preclude consumption by another’. (Ng and Ruger, 2011)	*‘* ***Global public goods*** provide a language by which economists of one market persuasion can convince economists of another that there is a sound rationale for a system of shared global financing and regulation’.	Public goods; disease surveillance; shared health rules; common goods.
4. Trade	‘International trade policies and agreements need to be placed within the context of protecting and promoting health and wellbeing’. (Amorim et al., 2007)	*‘* ***Trade*** can improve health through global market integration, economic growth and positive health externalities. However, present trade rules skew benefits towards more economically and politically powerful countries; and evidence of negative health externalities demands careful *a priori* assessments of trade treaties for their health, development and human rights implications’.	Trade; globalisation; economic growth; liberalisation.
5. Human rights	‘Health as a human right moves health provision from a discretionary charitable activity to a human entitlement or global citizenship right, adding moral force to actions and appeals to help the poor. Advancing health as a human right is consistent with advancing other human rights, such as civil and political rights imbued in democracy (believed to have positive influence on health), as well as social and economic rights’. (Ng and Ruger, 2011)	*‘* ***Human rights***, though weak in global enforcement, have advocacy traction and legal potential within national boundaries. Such rights do not resolve embedded tensions between the individual and the collective, an issue to which human rights experts are now attending’.	Human rights; democracy; justice; advocacy; collective rights; citing UN charter, Vienna declaration and so on.
6. Ethical/moral reasoning	‘Health has special importance to an individual’s experience of security or dignity . . . Health is both ‘intrinsically and instrumentally valuable’. But achieving it demands resources for capabilities. This immediately surfaces questions of *social justice* in how fairly (equitably) resources are allocated amongst peoples and, from a global health equity perspective, countries’. (Labonte and Gagnon, 2010)	*‘* ***Moral/ethical reasoning*** is suggested as a necessary addendum to the legalistic nature of human rights treaties. This need, in turn, has created scholarly momentum to articulate more rigorous argument for a global health ethic based on moral reasoning. Competitors for such an ethic range from a liberal theory of assistive duties based on ‘burdened societies’ in need, to cosmopolitan arguments that emphasise minimum capabilities needed for people to lead valued lives, to more recent arguments for a new ethic of relational justice based on cosmopolitan and human rights theories’.	Morals; ethics; dignity; social justice.

By analysing key policy documents in the regions using these policy frames, we seek to assess how these regional organisations that represent different populations in the world justify common positions on health. Critical discourse analysis is useful for this purpose since it is both normative and explanatory. It is normative in that it does not only describe and evaluate existing realities, but it equally seeks to explain them by assessing if and how they match up with values that are taken to be fundamental. It is explanatory because it shows how policy documents are the effects of mechanisms or forces explained by the frames and which we seek to unpack and test (Fairclough, 2013). This method is clearly useful in the case of the study of regional organisations, given their role in reaching consensus among their member states, which is expressed in the policy documents. In order to do this, we conduct a review of existing grey literature and scholarly work that address regional integration and health according to our policy frames. Moreover, we chose to analyse policy documents since these are the concrete manifestations of consensus at the regional level. We are aware that these documents may not explain the complexity result of the heterogeneity within regions, yet through analysing these policy documents, we can assess how they choose to interact with the outside world as a unit. Table 1 explains our approach to these policy frames as well as examples of key words used for the analysis.

The inclusion criteria for our regional bodies are as follows: (1) regional bodies that encompass the largest population in the region, (2) regional bodies that have a mandate in addressing health issues or have developed activities in health, and (3) access to primary documents. The authors’ expertise in the ASEAN, the EU, the SADC and the UNASUR was also taken into account.

Within these regional bodies, we conduct a search of their main policy documents. These include the following: (1) regional charters, (2) health protocols/working plans, (3) resolutions, and (4) position papers at international conferences. Other sources included: official websites as well as published interviews and commentaries. We searched for documents published between 2000 and 2014 on relevant databases. This time period allowed us to look at the evolution in thinking on health diplomacy in these regional bodies during the MDG period and towards a post-2015 scenario. Searched databases included EMBASE, PubMed and Google Scholar.

However, the study is burdened by two main limitations. First, although we have made efforts to complement online institutional grey literature through correspondences with anonymous key informants in the respective regional organisations, it is probable that key texts eluded our attention. In addition, published texts may reflect a lapsed reality and may not capture current discussions around health in these regional organisations. This is the case for example of the SADC, where texts have a 2-year embargo period after which they are freely available to the public. Concerning the use of critical discourse analysis, it has been argued that the analysis responds to the researchers’ interpretation of meaning, which may be different to that intended by the authors of the texts (Tenorio, 2011). This is related to the issue of understanding the context within which the documents are framed.

Furthermore, in this approach, we do not suggest that regional organisations are static bodies with one point of view towards health. Indeed, health involves a multiplicity of issues from the economic, political and medical areas (Kleinman, 2010), and this diversity of interests is compounded by the distinct health positions member states have within a regional organisation. In this sense, these organisations may approach health internally in a different manner than how they present their positions to other regions or at the global level. In our findings, we present the most common approaches to health by these organisations and assess the predominance of one position according to the relevance of the policy documents and in some cases, the concrete actions taken to implement such approaches.

## Four different context-specific health policy frames

The four regional organisations we analysed were founded in different temporal contexts. While a majority of them (with the exception of the UNASUR) emerged primarily as economic cooperation entities, they have developed varying mandates in health. It should also be recognised that they represent a wide range of development levels both within and between them. These issues are reflected in the policy frames identified, as well as in the literature available for each of these regions. The EU has a relatively wider spectrum of documents on health. This can partly be explained by its involvement in domestic regional issues, as well as cooperation with other countries outside of Europe. We identify different preferred policy frames in the four regional bodies with the ASEAN leaning more (not exclusively) towards a securitisation of health approach. The EU considers health as an overarching issue. The SADC prioritises health as a development issue. The UNASUR focuses on health and equity as an ethical imperative.

### ASEAN: A region emphasising development and (increasingly) the securitisation of health

ASEAN was formed in 1967 as a grouping of five countries in Southeast Asia. Over the years, it has expanded to include 10 countries in the region.^2^ Among its overall aims are accelerating economic growth, promoting regional peace and stability, encouraging collaboration around common interests, promoting Southeast Asian studies and cooperating with other international and regional organisations (ASEAN, 2014a). In health, ASEAN has four main priorities: access to health care, promotion of healthy lifestyles, improving capability to control communicable diseases, and ensuring a drug-free ASEAN (2000).

Our documentary search for ASEAN resulted in few available documents addressing health and foreign policy. While ASEAN leaders often consider that the basis for ASEAN Cooperation in Health was already implicit in the Bangkok Declaration in 1967 and the Declaration of ASEAN Concord in 1976, public health and regional cooperation in this area only emerged high on the ASEAN agenda in 1980 when for the first time ASEAN health ministers decided to meet regularly and ‘to strengthen and coordinate regional collaboration in health among ASEAN countries’.^3^ Since then, and notably after the severe acute respiratory syndrome (SARS) epidemic, health became a significant issue for the ASEAN and an important dimension of several important documents.

These include the Healthy ASEAN 2020 plan (2000); the declaration of the eighth ASEAN health ministers meeting, Unity in Health Emergencies (2006a); the 13th ASEAN Regional Forum declaration (2006b); the 12th ASEAN Regional Forum declaration (2005) and the ASEAN Socio-cultural community (ASCC) Blueprint (2009). After analysing these documents, three main policy frames emerge within the ASEAN region: a human rights, development and security approach (see Table 2).

**Table 2.** ASEAN health and foreign policy frames.

Title	Year published	Identified policy frame(s)	Example	Source
Healthy ASEAN 2020	2000	Human rights	*‘Health as a fundamental right of our peoples’* (p. 1)	http://www.asean.org/communities/asean-socio-cultural-community/item/declaration-of-the-5th-asean-health-ministers-meeting-on-healthy-asean-2020-28-29-april-2000-yogyakarta-indonesia
		Development	*‘Health shall be at the centre of development’.* (p. 2)	
Declaration of the eighth ASEAN Health Ministers Meeting, Unity in Health Emergencies, 21 June 2006, Yangon	2006	Security	*‘A stable and secure ASEAN Community can be realised only when our peoples enjoy optimum health’.*	http://www.asean.org/communities/asean-socio-cultural-community/item/declaration-of-the-8th-asean-health-ministers-meeting-asean-unity-in-health-emergencies-yangon-21-june-2006
13th ASEAN Regional Forum, Kuala Lumpur, Malaysia, 28 July 2006	2006	Security	‘*Avian and pandemic influenza and other infectious diseases such as HIV/AIDS posed significant potential security threats to the countries in the region*’. (p. 459)	http://aseanregionalforum.asean.org/files/ARF-Publication/ARF-Document-Series-1994-2006/13_KualaLumpur2006.pdf
12th ASEAN Regional Forum, Vientiane, Lao PDR, 29 July 2005	2005	Security	‘*The Ministers shared their concerns about highly pathogenic avian influenza and recognised its growing threat to both human and animal health as well as to the broader security of the region’.* (p. 358)	http://www.mofa.go.jp/region/asia-paci/asean/conference/arf/state0507.html
ASEAN Socio-cultural community (ASCC) Blueprint	2009	Security	‘Consolidate, further strengthen and develop regional cooperative arrangements through multisectoral and integrated approaches in the prevention, control, preparedness for emerging infectious diseases’	http://www.asean.org/archive/5187-19.pdf
			‘Ensure that stockpile of antivirals and Personal Protective Equipment (PPE) is maintained at regional level for all member states’. (p. 9)	
		Development	*‘ASEAN is committed to enhancing the wellbeing and the livelihood of the peoples of ASEAN through alleviating poverty, [ . . . ] and addressing health development concerns’.* (p. 6)	

The human rights argument presented in the Healthy ASEAN 2020 document (ASEAN, 2000) was recently confirmed in the ASEAN Human Rights Declaration that reiterates that ‘every person has the right to the enjoyment of the highest attainable standard of physical, mental and reproductive health, to basic and affordable health-care services and to have access to medical facilities’ (ASEAN, 2012). Nonetheless, such a right is limited to specific situations as the declaration stipulates that

the exercise of human rights and fundamental freedoms shall be subject only to such limitations as are determined by law solely for the purpose of securing due recognition for the human rights and fundamental freedoms of others, and to meet the just requirements of national security, public order, public health, public safety, public morality, as well as the general welfare of the peoples in a democratic society. (ASEAN, 2012)

However, these restrictions open the doors to many possibilities to limit the rights of sick people in the name of ‘national security’. This is more so as governments may place arbitrary, disproportionate and unnecessary restrictions on the human rights of these people.

The Healthy ASEAN 2020 document also includes the development argument. It expresses the position that health should be at ‘the centre of development’ (ASEAN, 2000). Here, the ASEAN considers health as an engine for social development and consequently the prerequisite for the wellbeing of every ASEAN citizen. Such a link between ‘health’ and ‘social development’ is especially visible in the ASCC Blueprint (2009) which places health under the heading of ‘social welfare and protection’. It equally suggests that access to adequate and affordable health care and promotion of healthy lifestyle be ensured in order to enhance the wellbeing and the livelihood of the peoples of the ASEAN (2009). In this context, collaboration among the ASEAN member states on health promotion, lifestyle and risk factors of non-communicable diseases as well as sharing of best practices on primary health care infrastructure development are highly encouraged by the ASEAN Secretariat.

When it comes to dealing with communicable diseases, the ASEAN has, however, adopted another policy frame: the security frame. Following the global trend of regarding communicable diseases a threat to national/regional/global security, the ASEAN (2006a), which in general considers health a prerequisite for regional stability and security has on many occasions, presented challenges such as HIV/AIDS and avian influenza as significant security threats to member states (ASEAN, 2005, 2006b).

The impact of such ‘security’ framing of a health issue is particularly obvious in the decision of members of ASEAN to enhance their commitments to cooperate in addressing emerging diseases; to develop regional policies to face potential pandemics, notably within the framework of the ASCC Blueprint (2009) and the ‘ASEAN Medium Term Plan on Emerging Infectious Diseases (EID) (2011–2015)’.^4^ They also aspire to create regional mechanisms of health cooperation such as the ASEAN +3 Partnership laboratories (APL) or the ASEAN +3 Field Epidemiology Training Network (FETN).

Furthermore, improving the capacity to control communicable diseases is clearly the most developed area in terms of regional cooperation in the ASEAN. Members of the ASEAN often express their interest in supporting these efforts despite limited intra-regional financial support. At the same time, this is the sector that attracts the most financial support from external partners (for example from the World Health Organisation [WHO], the EU, the United States Agency for International Development [USAID], the AusAid and the Japan International Cooperation Agency [JICA]). Regional cooperation and international partnership with the ASEAN on other issues, such as addressing non-communicable diseases, is lower.

Framing a health issue such as communicable diseases as a ‘security issue’ has motivated the ASEAN members to strengthen their cooperation in this domain. In addition, it has led the ASEAN to enhance its cooperation with global health partners such as the United States and the EU in order to strengthen regional/national pandemic preparedness (Rollet, 2015).

### European Union: Proposing health in all policies at the regional level

The EU is an economic and political partnership that dates to the 1950s with six founding members: Belgium, France, Germany (West), Italy, Luxembourg and the Netherlands that has now expanded to 28 European countries^5^ covering most of Europe (European Union, 2014). In the area of health, the EU’s objectives are centred on fostering good health in an ageing Europe, protecting citizens from health threats and supporting the generation of dynamic health systems and new technologies (European Commission, 2007).

The EU has a wealth of documentation on health, given its important investment in this area both within the Union as well as abroad. Our literature review draws from 10 main documents addressing health as a foreign policy issue (see Table 3). Health was primarily presented within the development (European Commission, 2010c; European Commission, 2007), human rights (Council of the EU, 2010a; European Commission, 2010c), security (European Commission, 2010c; European Commission, 2007) and GPG (European Commission, 2010c) frames. However, our analysis also uncovered another frame that had not initially been described by Labonte and Gagnon (2010), which sees health as an overarching intersectoral issue (European Commission, 2007; Treaties of Nice, 2003/Lisbon 2009).

**Table 3.** EU health and foreign policy frames.

Title	Year published	Identified policy frame(s)	Example	Source
Treaty of Nice	2003	Overarching/intersectoral issue	‘A high level of human health protection shall be ensured in the definition and implementation of all Community policies and activities’. (art.152 [TN], art. 168 [TL])	http://europa.eu/legislation_summaries/glossary/nice_treaty_en.htm http://eur-lex.europa.eu/legal-content/EN/ALL/;ELX_SESSIONID=v2s6JX8Yx2qcMyp8XrXpnm1ThJMRQ4ds8JydFZGQMFyrMppl0Npj!1263621023?uri=OJ:C:2007:306:TOC
Treaty of Lisbon	2009			
EU Health Strategy –‘Together for Health’ (2008–2013)	2007	Development	‘Health is important for the wellbeing of individuals and society, but a healthy population is also a prerequisite for economic productivity and prosperity. [ . . . ] Spending on health is not just a cost, it is an investment’. (p. 5)	http://ec.europa.eu/health-eu/doc/whitepaper_en.pdf
		Development	‘Health as an important element in the fight against poverty through health-related aspects of external development cooperation with low income countries’. (p. 6)	
		Overarching/intersectoral issue	‘Health is not an issue for health policy alone’.(p. 6)	
		Security	‘Pandemics, major physical and biological incidents and bioterrorism pose potential major threats to health. Climate change is causing new communicable disease patterns. It is a core part of the Community’s role in health to coordinate and respond rapidly to health threats globally and to enhance the EC’s and third countries’ capacities to do so. This relates to the Commission’s overall strategic objective of Security’. (p. 3)	
‘Communication on The EU Role in Global Health’, European Commission (2010c) 128 final	2010	Development	‘Health is influenced by social, economic and environmental factors which are increasingly influenced by globalisation. Globally, improved health also depends on greater social justice’. (p. 2)	http://eur-lex.europa.eu/legal-content/EN/TXT/PDF/?uri=CELEX:52010DC0128&from=EN
		Development	‘Health as an essential objective within the MDG framework [ . . . ] Health is a critical element to reduce poverty and promote sustainable growth. [ . . . ] Special attention is given to poverty-related diseases and to the crisis of human resources for health’. (p. 4)	
European research and knowledge for global health SEC (2010) 381 final	2010	Global public goods (GPG)	‘Control (possibly eradication) of communicable diseases is one clear example of a GPG. It would benefit everyone, in poorer and richer countries alike and in present and future generations . . .’ (p. 4)	http://ec.europa.eu/health/eu_world/docs/swd_sec2010_381_en.pdf
		GPG	‘Research and development to generate medical knowledge is another example of a public good that is inherently global in nature’. (p. 4)	
Contributing to universal coverage of health services through development policy SEC(2010) 382 final	2010	Human rights	‘The EU is committed to protecting and promoting health as a human right for all’. (p. 11)	http://aei.pitt.edu/37948/1/SEC_%282010%29_382_final.pdf
Global health – responding to the challenges of globalisation SEC(2010) 380 final	2010	Security	‘Health can be a good entry point to initiate dialogue across borders, thus contributing to building trust between parties’. (p. 24)	http://ec.europa.eu/health/eu_world/docs/swd_sec2010_380_en.pdf
		Development	‘Health does not only contribute to economic growth, it also represents a major provider of employment in the EU and globally’. (p. 12)	
Council conclusions on the EU role in Global Health, 3011th Foreign Affairs Council Meeting	2010	Human rights/Development	‘Health is central in people’s lives, including as a human right, and a key element for equitable and sustainable growth and development, including poverty reduction’. (p. 1)	http://www.consilium.europa.eu/uedocs/cms_Data/docs/pressdata/EN/foraff/114352.pdf
Investing in health. (2013)	2013	Development	‘Health is a value in itself. It is also a precondition for economic prosperity. People’s health influences economic outcomes in terms of productivity, labour supply, human capital and public spending’. (p. 1)	http://ec.europa.eu/health/strategy/docs/swd_investing_in_health.pdf

This frame is clearly expressed in the Nice and Lisbon treaties where they promote the ‘Health in all policies’ (HIAP) approach. The rationale here is the recognition that social, environmental and economic factors act as determinants of health. Indeed, the EU defines HIAP as a policy strategy that targets the key social determinants of health through integrated policy responses across relevant policy areas with the ultimate goal of supporting health equity. In this sense, this frame combines the conceptualisations of health as GPG – that is, goods which are not diminished by use and available to all – and as a driver of development, yet it goes further by including how health has an effect on and is affected by all aspects of society.

This conceptualisation of health has been widely documented in the global health discourse, rooted in Alma-Ata declaration (WHO, 1978) that underlined the need for an ‘intersectoral approach to health’. This was followed by the Ottawa Charter for health promotion proposing the development of ‘healthy public policies’ (WHO, 1986). This overarching intersectoral frame is further confirmed in the EU health strategy (2008–2013), an important document given that its principles and objectives will remain valid for the next decade in the context of Europe 2020; stating that ‘health is not an issue for health policy alone’. This view is also presented in the ‘Council conclusions on equity and health in all policies: solidarity in health’ document (Action for Global Health, 2012). As previously explained, the view that health is an intersectoral issue that should be addressed by all sectors is related to the GPG and development frames. This thinking is embedded in the main EU policy documents and guides their investments within the region and to other non-member states.

To a greater degree than the other regions analysed in this article, the EU provides support for health for its member countries as well as other countries through official development assistance (ODA). Our analysis reveals that the EU is using the ‘development’ policy frame to explain the value of addressing health issues externally by providing ODA to developing countries, as well as intra-regionally.

For example, we identify the development frame in the EU health strategy (2008–2013) that places health as an engine of economic growth and prosperity at the EU level. The Europe 2020 economic-growth strategy also presents health as central to development by keeping people healthy and active, through innovations in the health sector, generating jobs and financing rising health costs for an ageing population (European Commission, 2012).

In its approach to non-member countries, the EU considers ODA for health as a tool to fight poverty, support development and reach the MDGs. In this approach, the EU considers that investments in non-member states have the potential to open new economic opportunities and markets to the region. This is reflected in the percentage of ODA allocated to health, which represented 7.2% of the overall budget in 2010 (Action for Global Health, 2012).

Likewise, the Communication on the EU’s role in Global Health (European Commission, 2010c) underlines the social determinants of health and emphasises the direct link between health and the distribution of wealth, opportunities and privileges within societies. Within this document, it seems that the EU’s priority in terms of global health issues are poverty-related diseases seen as the major cause as well as the consequence of poverty in developing countries and the issue of human resources for health. Such specific attention reflects the focus made by the United Nations (UN) General Assembly Resolution on global health and foreign policy (United Nations, 2009) on these two topics.

In line with these policy commitments, the EU has funded research on poverty-related diseases through its Research Framework Programmes (FP7 and Horizon 2020). It also launched a partnership with Gates Foundation in 2013 to develop drugs, vaccines and diagnostics in this domain and has provided support to developing countries as well as international organisations to combat such health challenges. Concerning the second priority, it has been underlined that evidence of any concrete impact of the EU’s commitment to reduce migration of health workers from Africa notably has so far been poor.^6^

The frame of health as GPG was less common. The GPG argument was identified in the ‘European research and knowledge for global health’ (European Commission, 2010d) document where the EU considers control of transmissible diseases as GPG, that is, goods which are not diminished by use and are available to all.

The EU also considers medical research as a GPG. However, this document also recognises that the end-use of medical knowledge when it is embodied in a tangible good such as a drug often remains excludable and not available for all. Such an approach is the main rationale for the EU framework programme for health and currently, Horizon 2020 (European Commission, 2014). Through these initiatives, the EU aims to provide public funding for public knowledge in health in order to improve global health. The EU does this by subsidising research directly and providing effective incentives for private engagement in research.

Other less prevalent frames that emerged in our analysis were the security and human rights frames. The EU has witnessed a progressive securitisation of health since 2001 (i.e. 11 September 2001 and the subsequent anthrax scare), first in the context of its fight against bioterrorism and then in the context of SARS, H5N1 and H1N1, which engender the inclusion of (re)-emerging communicable diseases as a health security issue.^7^ Such framing has certainly helped through the investment of funds to create the EU Health Security Committee in 2001 and the European Centre for Disease Prevention and Control (CDC) in 2004, to develop research on health security in the Research Framework Programme 7 (FP7) context and to strengthen EU preparedness and response to such threats.

Moreover, this security frame was prevalent in the EU health strategy (2008–2013) and is further confirmed in the Commission Staff Working document on ‘Health Security in the European Union and Internationally’ (European Commission, 2009) and the ‘Commission Staff Working document on lessons learnt from the H1N1 pandemic and on health security in the European Union’ (European Commission, 2010b).

However, in the document ‘Global health – responding to the challenges of globalisation’, health is also seen as an entry point for dialogue with other nations (European Commission, 2010a). This might have been inspired by the ‘Health as a bridge for peace’ approach formally accepted by the 51st World Health Assembly (WHA) in May 1998. The approach supports health workers in delivering health in conflict and post-conflict situations and simultaneously contributes to peace building (WHO, 2014). Such an approach rests notably on the case of the South-East Europe Health network which helped to build bridges across hitherto hostile communities which then faced common problems following the conflicts in the Balkans during the 1990s (Dehnert and Taleski, 2013).

The human rights frame was less common. It was identified in ‘Contributing to universal coverage of health services through development policy’ (European Commission, 2010e) and ‘Investing in health’ (European Commission, 2013). In the latter document, the EU is actually supporting and promoting human rights beyond its borders. This right to access to health services is deeply rooted in the Alma-Ata Declaration (WHO, 1978) and the EU Charter of Fundamental Rights (European Union, 2000) stipulating that ‘ Everyone has the right of access to preventive health care and the right to benefit from medical treatment under the conditions established by national laws and practices’ (Art.35).

This frame is also consistent with the EU’s human rights policy that encompasses civil, political, economic, social and cultural rights considered as determinants of health as well as with the right to health projects implemented by the EU within the framework of its European Instrument for Democracy and Human Rights. Finally, defining health as a human right is different to the public goods frame. As Labonte and Gagnon (2010) explain, it possesses advocacy traction and above all, legal potential within national boundaries and beyond.

### SADC: The important impact of HIV/AIDS in framing health as a development issue

The SADC is a regional economic community that was established in 1992 and is comprised of 15 members.^8^ Among its goals are achieving regional integration and poverty eradication in the region through economic development, while ensuring peace and security for its member states (SADC, 2014b). Health is integrated within the context of social and human development, poverty and food security. Moreover, given the importance of HIV/AIDS in the region, it is addressed as a stand-alone cross-cutting issue within the SADC (2014a). The importance of HIV/AIDS in the region is also reflected in our results.

Six documents in total are identified as the most important documents addressing health in SADC.^9^ In these documents, the most commonly found policy frames in the analysis centred on development, trade and human rights arguments (see Table 4). Of these frames, development was the most frequent argument for health in foreign policy. Given the economic situation in the region, how SADC defines development is different from the ASEAN and the EU cases. The SADC seems to address development as the reduction of poverty rather than the increase of wealth, which are in themselves different approaches. We see that the SADC health protocol (1999) starts by reaffirming that a healthy population is a prerequisite for sustainable development. The text was adopted during a period regarded as the acme of efforts to ensure better access to affordable health care to HIV/AIDS patients in many countries in the region.

**Table 4.** SADC health and foreign policy frames.

Title	Year published	Identified policy frame(s)	Example	Source
SADC Health Protocol	1999	Development	*‘A healthy population is a prerequisite for sustainable development and increased productivity in member states’. (Recital 3)*	http://www.sadc.int/documents-publications/show/804
		Trade	*‘States shall cooperate in assisting one another in the production, procurement and distribution of affordable essential drugs’. (Article 29b)*	
		Human rights	*Among the goals of the protocol is to ‘develop common strategies to address health needs of women, children and other vulnerable groups’. (Article 3g)*	
Regional Indicative Strategic Development Plan	2003	Development	*‘In the area of health, the main goal of integration is to attain an acceptable standard of health for all SADC citizens and to reach specific targets within the objective of “Health for All” in the 21st century by 2020 in all Member States through the primary health care strategy’. (recital 3)*	http://www.sadc.int/files/5713/5292/8372/Regional_Indicative_Strategic_Development_Plan.pdf
Maseru Declaration on the Fight against HIV/AIDS in the SADC Region	2003	Development	*‘The HIV/AIDS pandemic is reversing the developmental gains made in the past decade and is posing the greatest threat to sustainable development of the region due to loss of the most productive individuals . . . ’*	http://www.sadc.int/files/6613/5333/0731/Maseru_Declaration_on_the_fight_against_HIVand_AIDS2003.pdf.pdf
SADC Declaration on Poverty Eradication and Sustainable Development	2008	Development	*‘They resolve to step up actions to ensure access to health services including primary health care and to increase efforts to combat HIV/AIDS’. (Clause 2(V))*	http://www.sadc.int/files/8013/5340/5415/Declaration_on_Poverty_Eradication_ann_Sustainable_Development2008.pdf.pdf
Sexual and reproductive health business plan for the SADC region 2011–2015	2012	Development	Among the main goals of the plan is to *‘accelerate the attainment of healthy sexual and reproductive life for all SADC citizens’. (p. 3)*	http://www.sadc.int/files/3613/5293/3504/SADC_Sexual_and_Reproductive_Health_Business_Plan_2011-2015.pdf
SADC Strategy for Pooled Procurement of Essential Medicines and Health Commodities 2013–2017	2012	Development	SADC’s common agenda includes promotion of *‘sustainable and equitable economic growth and socio-economic development that will ensure poverty alleviation with the ultimate objective of its eradication, enhance the standard and quality of life of the people of Southern Africa and support the socially disadvantaged through regional integration’. (p. 1)*	http://www.sarpam.net/wp-content/uploads//images+docs/4-stakeholders/b-sadc/SADC-Pooled-Procurement-Strategy-210213.pdf
		Trade	*‘Among the Priorities of the SADC Pharmaceutical Business Plan is to address TRIPS to ensure that essential medicines are available, safe, effective and affordable’. (p. 2)*	

TRIPS: trade related aspects of intellectual property

The ‘Regional indicative strategic development plan’ (SADC, 2003a) also approaches health as a means to achieve greater development, and it refers to the goal of primary health care for all by 2020 as espoused in the Alma-Ata Declaration (WHO, 1978). Furthermore, the ‘SADC strategy for pooled procurement of essential medicines and health commodities’ (SADC, 2012:1) describes how greater access to medicines can support the goal of sustainable growth and poverty eradication.

The ‘Maseru declaration on the fight against HIV/AIDS in the SADC region’ (SADC, 2003b:2 and Recital 8) makes the link between health, particularly HIV/AIDS with development by exposing the threat to the economies of the region resulting from the deaths and demise of productive individuals. This is further stated in the ‘SADC declaration on poverty eradication and sustainable development’ where member states reaffirm their commitment to achieve key international goals such as the MDGs and commit to increasing access to services and combating HIV/AIDS.

Trade was an important frame that was also frequently linked to HIV/AIDS. The SADC Health Protocol (1999) explicitly states the commitment to support member states in the provision of affordable essential drugs. This section was adopted in a tense atmosphere given the debates that ensued at the time, on access to affordable medicines. This included a context where a serious battle was taking place between the government of South Africa and pharmaceutical companies due to changes in South African legislation for antiretrovirals. All these tensions culminated in the failed World Trade Organisation (WTO) Seattle Ministerial of November 1999 (T’Hoen, 2002).

The access to affordable medicines debates and struggles especially in Southern Africa were specifically articulated in human rights terms. Groups such as Treatment Action Campaign and Section 27 used this approach. Furthermore, this struggle is compounded by the fact that some member states continue to rely on donor aid for procurement of essential medicines. In order to address this, the ‘SADC strategy for pooled procurement of essential medicines and health commodities’ (2012) was adopted. It is stated in this strategy that one of the priorities of the plan is to address limitations in the WTO sanctioned Agreement on Trade Related Aspects of Intellectual Property (TRIPS) in order to have better access to safe, effective and affordable drugs and vaccines.

In as much as the right to health and to drugs was frequently presented in human rights terms, there is also an inclination to dilute some of the gross inequities in terms of access to affordable health care marked in Southern Africa by a legacy of apartheid and chasms in the topography of universal health care in the region by focussing on vulnerable populations. In countries like Botswana, Namibia, South Africa and Zambia, this is epitomised by publicly-funded free access to specific medicines to citizens and residents of disadvantaged communities.

### UNASUR: A focus on ethical reasoning and human rights

The UNASUR was founded in 2008 as an organisation seeking South American regional integration in the areas of energy, education, health, environment, infrastructure security and democracy. It currently includes 12 member countries^10^ (UNASUR, 2014). In the area of health, the UNASUR’s strategic objectives prioritise vulnerable and excluded populations, as well as populations in high-risk areas. There is also an important focus on social determinants of health and health promotion (UNASUR, 2009). This was generally regarded as the result of the shift towards a more left-wing political and social agenda (Riggirozzi, 2014).

Six documents are identified as the most important in the area of health for the UNASUR with documents frequently cross-referencing each other. The most common frames are ethical reasoning, development, human rights, public goods and security (Table 5).

**Table 5.** UNASUR health and foreign policy frames.

Title	Year published	Identified policy frame(s)	Example	Source
UNASUR constitutive treaty	2008	Ethical reasoning	*‘Universal access to social security and health services’ (Art. 3, p. 3)*	http://www.unasursg.org/uploads/0c/c7/0cc721468628d65c3c510a577e54519d/Tratado-constitutivo-english-version.pdf
Resolution 9/2011. Declaration of the UNASUR South American Health Council on strengthening national health systems.	2011	Development/ethical reasoning	*‘Health is an indispensable condition to achieve economic and social development of the countries. The States have the responsibility to guarantee equity in health as a means to improve the living standards of the South American citizens and sustainable development of the countries’. (p. 1)*	http://www.isags-unasur.org/uploads/biblioteca/1/bb%5B53%5Dling%5B3%5Danx%5B112%5D.pdf
ISAGS 3-year working plan 2012–2015	2011	Human Right/Public Good/Development	*‘Health as*: A fundamental right of a human being and of society and a vital component for human development; Driving force of regional integration. Central component of social protection and harmonious social development. Space for the reduction of existing asymmetries among countries. A public good that regards the joining of society and space as vital for citizen participation’.	http://www.isags-unasur.org/uploads/biblioteca/1/bb%5B47%5Dling%5B3%5Danx%5B297%5D.pdf
Constitution of the South American Council for Health No. 01/09-21/04/2009	2009	Human rights	*‘Health is a fundamental right . . . ’ (p. 2)*	http://www.ocai.cl/unasur-english.pdf
		Security	*Emphasis on the ‘development of the integrated actions of epidemiological and entomological alertness emphatically in the zones of border [sic]’. (Annex: p. 6).*	
UNASUR Salud Plan Quinquenal 2010–2015	2009	Human rights	*‘Health is a fundamental right for human beings and society and is a vital component of and for human development’.*	http://www.ins.gob.pe/repositorioaps/0/0/jer/rins_documentosunasur/PQ%20UNASUR%20Salud.pdf
		Development	*‘Health should be integrated in the greater concept of social protection and as such, should play an important role in harmonic social development’.*	
RINS (Network of National Institutes of Health)/UNASUR Plan Quinquenal 2011–2015	2010	Human rights	*‘Establish as a cross-cutting axis for development the recognition of the fundamental human rights of people as active actors in the actions of transformation in their societies, where the right to health is the dynamic axis of their people within the process of Southern American integration’.*	http://rins-unasur.org/index.php/documentos/cat_view/5-documentos-aprobados
		Development	*‘Establish policies for action that lead to the elimination of social differences, decrease the economic abyss that separates people and recognise and respect the differences of gender, policies, environment and cultural’.*	

The position of UNASUR on health can be clearly identified in the ‘UNASUR constitutive treaty’ (2008) that discusses the need to promote universal access to social security and health services. It points towards an ethical reasoning or basis of health in foreign policy. This frame was also identified in resolution 9/2011 that provides for the responsibility of the states in guaranteeing equity and improving the living standards of their citizens (UNASUR, 2011). Furthermore, the argument of health as a fundamental human right is identified in the majority of the documents, primarily in the ‘Five Year UNASUR Health Work Plan (2010–2015)’, the UNASUR’s main guiding document for health activities (UNASUR, 2009).

Health as a driver of development also emerges as an important frame in several documents. Health is considered to be a crucial condition for economic and social development in resolution 9/2011. This is also the case in the periodic working plan of the South American Institute of Governance in Health (ISAGS). Related to the ethical approach, health is regarded as a means to reach equity and ‘harmonious development’ (i.e. ISAGS, 2011; RINS, 2010; UNASUR, 2009), which reflects the importance UNASUR gives to sustainable development. Moreover, this convergence of social, environmental and health policies reminds us of the EU’s proposal of HIAP. Nonetheless, the UNASUR does not go as far in stating this within its approach to health.

The importance of health as a security issue is also a frame that emerges in several documents usually around the idea of the development of an ‘epidemiological shield’ in the region. This is a security issue since it entails the protection of citizens by stopping the spread of new diseases from neighbouring states and migration. This frame was strongly reflected in the ‘Constitution of the South American Council for Health’ that calls for collaborative action on addressing epidemiological and entomological threats, particularly in the border regions (UNASUR, 2009a). This reflects the work of the UNASUR in supporting trans-border agreements between Argentina, Brazil and Paraguay to address dengue fever, for example (Pan American Health Organisation [PAHO], 2010).

Within our analysis we find some references to health as a ‘public good’, most notably in the ISAGS working plan but this is not a term frequently used (ISAGS, 2011). Health as a common point of convergence that can promote regional integration (ISAGS, 2011; RINS, 2010) seems to an argument that is repeatedly rehearsed.

The ISAGS 3-year working plan discusses access to medications but does not bring up trade issues (ISAGS, 2011). The limited discussion around trade may reflect the countries’ limited involvement in production (with the notable exceptions of Brazil and to an extent, Venezuela) and their focus on access to health services as an issue of social justice and not commerce.

Although the evidence shows that the UNASUR may justify involvement in health in different manners, these documents still reiterate their clear stance on approaching health as a human right. This is also made explicit in their core policy documents such as the ‘UNASUR Constitutive Treaty’ (2008) and the ‘Five Year UNASUR Health Strategic Plan’ (2009b), among others. This means states should guarantee access to health for all in the same manner that they ensure other human rights. This is not only their internal outlook on health among member states but is also their stated external contribution to foreign policy debates as a regional bloc (Amaya et al., 2015).

## Discussion

The findings from our analysis of the four regional organisations demonstrate that there is no unified perspective on health shared by the organisations. Instead, they seem to utilise different policy frames to justify the type of foreign policy action they want to make at a given time. This can be explained by the multifaceted nature of health, which is likely to be approached from different standpoints. Nonetheless, our analysis demonstrates that each of these organisations seems to align themselves to one preferred policy frame.

In the case of the ASEAN, its struggle with re-emerging diseases such as influenza has led to increasing sympathy for a securitisation approach to health, with health considered a major factor for regional stability and security (ASEAN, 2007). The EU translates the debate around ‘HIAP’ to the regional level by describing how health should be tackled as an intersectoral issue. For SADC, health is presented as a driver for development. This can be explained by the important impact the HIV/AIDS epidemic has had on development in the region. The UNASUR also sees health as a driver of development but frames health and equity as ethical imperatives for its member states. Although both the SADC and the UNASUR view health as an important element to reduce poverty, the UNASUR places greater focus on addressing the social determinants of health, the economic and social conditions that influence differences in health status. For its part, the SADC addresses poverty through the attainment of the MDGs and reduction of HIV/AIDS, which affects a significant segment of the population. Moreover, it is important to note that beyond the emerging issues that orientate them to approach health from a preferred angle in these documents, the regions’ conceptualisations of health are primarily related to the differing contexts present in their member countries that respond to cultural approaches to health, the economic situation in the area and their relationship with other regions.

Interestingly, another study found that the wider literature on the African region shows that common themes that can be used to explain African diplomacy on health are as follows: liberation ethic, African unity and interdependence, and developmental foreign policy (Loewenson et al., 2014). In this case, the researchers focussed on the entire continent and did not exclusively review policy documents, which was this article’s chosen approach. This may explain some of the differences in the findings. However, they coincide in finding a strong focus on development, which they assess as the result of the important influence of external funding on health. In this case, liberation ethic and unity are manifestations for a search for greater autonomy and self-reliance.

We used the Labonte and Gagnon (2010) framework to assess foreign policy positions on health at the level of regional organisations. Overall, the policy frames were useful in organising positions around health in foreign policy at the regional level. However, the analysis of these regional organisations’ documents demonstrates that while the ASEAN increasingly favours the traditional securitisation approach, regional organisations such as the UNASUR and the SADC do approach health from development and human rights standpoints.

The discrepancy in our findings from Labonte and Gagnon’s approach is not surprising, given that their original work analyses high-income countries, while we include regional organisations that are composed of a number of developing countries and hence have a greater stake in ensuring all member states develop to build a stronger union. Moreover, the EU documents (and to an extent the UNASUR’s) show that the policy frames proposed by Labonte and Gagnon (2010) do not account for views of health as a cross-cutting issue within foreign policy. The need for a policy frame to account for the involvement of actors from different sectors within foreign policy is also supported by the literature, which shows that involving environmental, social and economic governance is increasingly seen as the way forward in health policy (McQueen et al., 2002).

For regional organisations, such an approach would help strengthen the coherence of their policies and confirm the need for interagency cooperation at the regional level. It may also provide legitimacy to the organisation to act in the domain of health and lead to a diversification of resources (human, technical and financing) for health. On the other hand, this approach risks granting health an imperial position within policy when cross-sectoral action could also be useful for other domains such as education, the environment and human rights. Furthermore, given the EU’s involvement in development cooperation, such an approach may be incompatible with the policies of other governments, making any type of residual contribution problematic.

The interaction with other countries brings up the point of how these regional organisations negotiate their positions globally. While these regions have all presented statements at the WHA and to some extent, in the cases of the EU and the UNASUR,^11^ have successfully introduced proposals and action plans at this forum; other regional organisations besides the EU do not have a formal status and positions are brought forward by member states representing the regional blocs.

In the case of Africa, continent-wide initiatives have been more successful than sub-regional organisations in bringing positions forward at the WHA. For example, the continent was instrumental in the discussions around the preparation and adoption of the WHO Code of Practice on the International Recruitment of Health Workers, given the impact the migration of health care workers has on a large number of African countries (Dambisya et al., 2014). Yet, the convening power of sub-regional organisations such as the SADC has been lower.

The ASEAN’s swift position on addressing the SARS epidemic among their member states in 2003 was recognised by the global community as an example of effective international cooperation against a common disease threat (WHO, 2003). This also led to formal agreements between the ASEAN and the WHO (ASEAN and WHO, 2009) and the ASEAN plus 3 countries^12^ have recently brought forward common positions at the WHA level, for example, on Universal Health Coverage based on success reached in their member states (ASEAN, 2014b).

The EU and the UNASUR’s participation at the WHA, and to some extent the ASEAN’s, is important since it demonstrates that these regional organisations are increasingly becoming spaces of consensus, where the united voice of the countries expressing their concerns can both support the health situation of the member states that are lagging behind. Furthermore, the informal interactions between the EU and the UNASUR before voting in the WHA in order to seek common areas of interest and unified positions (whenever possible), demonstrates the value that the UNASUR has as a common voice on health matters for the South American region and their potential to formalise their role in the WHA (Riggirozzi, 2015). This could also open the pathway for other regional organisations to participate in this type of fora, advocating a distinctive regional perspective, even though only the EU currently is granted formal speaking/voting powers. However, competing conceptualisations and related interests of these regional organisations will likely have an impact at the moment of negotiating at instances such as the WHA, the UN General Assembly or the WTO.

These findings elucidate how regional organisations formally address health as a foreign policy issue, which explains their interaction with other outside bodies such as the WHO, the UN, donors and international civil society organisations, as well as within their member states. Identifying these approaches and the subsequent dynamics they lead to is an important contribution to the literature by elucidating regional organisations’ current role and potential to shape the global health governance scenario and pave the way for appreciable regional health diplomacy. This is an area that until now has been relatively unexplored within the health diplomacy and global governance literature.

Finally, we contribute to the debate on how health is being framed in foreign policy by researching regional organisations from four different continents and explaining the implications of these positions to health. We chose to analyse policy documents, given that these can be considered palpable manifestations of consensus generated in regional organisations between member states. Conducting further research in this area that includes the wider literature may provide new directions on how to maximise the impact of these organisations in the area of health.

## Conclusion

The policy frames of these regions are a clear expression of the different challenges that their populations currently face. This means that for now infectious diseases such as influenza and HIV/AIDS will continue to be a priority for the ASEAN and the SADC. The EU and the UNASUR will probably continue to favour a comprehensive approach to health to improve equity. Nonetheless, the approaching deadline for the MDGs and the discussion of the post-2015 agenda around the sustainable development goals (SDGs), which will likely include a general health goal to ‘ensure healthy lives and promote wellbeing for all at all ages’ (Open Working Group, 2014), may again galvanise the international community towards the same objectives.

Indeed, our analysis shows that regional organisations have recognised the importance of addressing health within their integration efforts as a way of strengthening their member states’ social response as well as its value for a resilient region. Health is also used as an entry point to juxtapose countries or regions. Understanding how others view health is key. This is also critical when interacting with donors in the case of the SADC region, for example, since it helps to encompass the main priorities in the area. Our findings support these interpretations. It analyses the salient views on health in these regions. This has implications both for the discussions in international relations, political science and global health of how common positions are created by consensus and then negotiated, as well as how health policies are prioritised. It also provides a clear basis on which to hold actors accountable for their commitments on health. Additionally, researching regional organisations’ approach to health can contribute to understanding the role of these within the wider global health governance arena.

The increasing role of regional organisations in health is clear. Their impact in influencing the post-2015 agenda and the rollout of the SDGs over the coming years will depend on their ability to harness their convening power and speak in a coherent voice on health matters.

## Notes

1. The NRA explains how as a result of the forces of globalisation, regions are the result of relations between state and non-state actors. Among other concerns, this approach seeks to explain the construction of regions and ‘regionness’, that how regions build identity and social cohesion.

2. Current ASEAN members include Brunei, Cambodia, Indonesia, Laos, Malaysia, Myanmar, the Philippines, Singapore, Thailand and Vietnam.

3. ASEAN, Declaration of the ASEAN Health Ministers on Collaboration on Health, 24 July 1980, Manila.

4. ASEAN, ASEAN Medium Term Plan on EID (2011–2015), Bangkok, 2011.

5. Current EU members include Austria, Belgium, Bulgaria, Croatia, Cyprus, Czech Republic, Denmark, Estonia, Finland, France, Germany, Greece, Hungary, Ireland, Italy, Latvia, Lithuania, Luxembourg, Malta, Netherlands, Poland, Portugal, Romania, Slovakia, Slovenia, Spain, Sweden and the United Kingdom.

6. Action for Global Health, Addressing the Global Health Workforce Crisis. Challenges for France, Germany, Italy, Spain and the United Kingdom, Brussels, January 2011.

7. European Commission, EU Health Strategy ‘Together for Health’ (2007), European Commission, ‘Health Security in the European Union and Internationally’ (2009) SEC(2009) 1622 final, European Commission, ‘Commission Staff Working document on lessons learnt from the H1N1 pandemic and on health security in the EU’ (2010b) SEC(2010) 1440 final. Also see Zylberman (2013: 145–149).

8. Current SADC member states include Angola, Botswana, Democratic Republic of Congo, Lesotho, Madagascar, Malawi, Mauritius, Mozambique, Namibia, Seychelles, South Africa, Swaziland, United Republic of Tanzania, Zambia and Zimbabwe.

9. Although the SADC health protocol was signed in 1999, it was included since it is the most important document guiding health policy at the SADC level and only entered into force in 2004.

10. Current UNASUR member states include Argentina, Bolivia, Brazil, Chile, Colombia, Ecuador, Guyana, Paraguay, Peru, Suriname, Uruguay and Venezuela.

11. In the case of the UNASUR, the organisation had an important role in the discussion of intellectual property rights on access to medicines.

12. ASEAN Plus 3 Three Countries include Brunei Darussalam, Cambodia, Indonesia, Lao People’s Democratic Republic (PDR), Malaysia, Myanmar, Philippines, Singapore, Thailand, Viet Nam, China, Japan and Republic of Korea (the latter 3 comprising the +3).
